# Evaluation of Coronary Artery Calcium Score (CACS) in Dipper and Non-Dipper Hypertensive Patients with Moderate and High Cardiovascular Disease Risks

**DOI:** 10.3390/medicina60121999

**Published:** 2024-12-03

**Authors:** Ahmet Cinar, Omer Gedikli, Muhammet Uyanik, Ozlem Terzi

**Affiliations:** 1 Department of Cardiology, Faculty of Medicine, Ondokuz Mayis University, 55270 Samsun, Turkey; drgedikli@hotmail.com (O.G.); muhammetuyanik@hotmail.com (M.U.); 2 Department of Public Health, Faculty of Medicine, Ondokuz Mayis University, 55270 Samsun, Turkey; ozlem.terzi@omu.edu.tr

**Keywords:** coronary artery calcium score (CACS), dipper hypertension, non-dipper hypertension, cardiovascular risk, atherosclerosis

## Abstract

*Background and Objectives*: Hypertension is typically classified into two main groups, “dipper” and “non-dipper”, based on nocturnal blood pressure decline. The coronary artery calcium score (CACS) is an essential biomarker used to assess the presence and severity of coronary artery disease (CAD). This study aims to demonstrate the relationship between CACS and hypertensive patients with moderate-to-high cardiovascular disease (CVD) risk classified as either dipper or non-dipper. *Participants and Methods*: A total of 167 patients with moderate-to-high CVD risk were divided into two subgroups: 95 patients with dipper hypertension (HT) and 72 with non-dipper hypertension. CACS was measured using coronary computed tomography angiography. *Results*: In the dipper HT group, there were 60 females (63.2%) and 35 males (36.8%), whereas the non-dipper HT group included 28 females (38.9%) and 44 males (61.1%) (*p* = 0.002). The mean age was 57 in the dipper HT group and 62 in the non-dipper HT group (*p* = 0.011). The mean CACS was 93 in the non-dipper HT group and 10 in the dipper HT group (*p* < 0.001). A history of coronary artery disease was more common in the non-dipper HT group (*p* = 0.003). Smoking prevalence was higher in the non-dipper HT group (31 patients, 43.1%) compared to the dipper HT group (25 patients, 26.3%) (*p* = 0.023). Correlation analysis showed that CACS was positively correlated with age, BMI, and HbA1c and negatively correlated with eGFR. Higher CACS values were also observed in males and patients with a history of coronary artery disease, diabetes mellitus, and hyperlipidemia. In univariate analysis, age, male sex, smoking, CAD, CACS, and elevated creatinine were identified as significant risk factors for non-dipper HT (*p* < 0.05). However, in multivariate analysis, only CACS emerged as a significant independent risk factor (*p* = 0.001), while other variables were not significant (*p* > 0.05). The area under the curve (AUC) for CACS was 0.759, indicating statistically significant and excellent discriminative capability (*p* < 0.001, 95% CI: 0.680–0.839). *Conclusions*: It was concluded that non-dipper hypertension is associated with higher CACS and indicates a higher cardiovascular risk for this group.

## 1. Introduction

Hypertension is a common and significant global health concern, recognized as one of the most important risk factors for cardiovascular diseases (CVDs). The circadian rhythm of blood pressure demonstrates distinct patterns, represented by the “dipper” and “non-dipper” phenotypes, which account for two major variations in blood pressure behavior. In dipper hypertension, blood pressure typically decreases by 10–20% during nocturnal sleep, a phenomenon generally associated with a better prognosis and lower cardiovascular risk. Conversely, non-dipper hypertension is characterized by the absence of this nocturnal decline or only a minimal reduction in blood pressure during the night (<10%). Non-dipper hypertension is linked to a higher cardiovascular risk, particularly in the progression of atherosclerosis, a major pathological process underlying cardiovascular events such as myocardial infarction and stroke [1].

The circadian pattern of blood pressure is integral to vascular health; the dipper pattern supports physiological processes contributing to vascular repair and homeostasis. This is primarily due to the reduction in arterial stiffness and vascular tone that typically occurs at night, allowing the endothelium to recover and repair itself. In contrast, the absence of the dipper pattern in non-dipper hypertension leads to sustained high blood pressure during the night, contributing to ongoing endothelial damage, increased arterial stiffness, and the acceleration of atherosclerotic processes. The lack of nocturnal blood pressure decline in non-dippers may also result in aberrant vascular remodeling, further exacerbating the progression of atherosclerosis and its clinical outcomes [1].

The relationship between blood pressure patterns and atherosclerosis is well-documented, with studies showing that non-dipper hypertensive patients tend to have higher coronary artery calcium scores (CACSs), a marker of subclinical atherosclerosis [2,3]. Elevated CACS is a well-known prognostic indicator for cardiovascular events and mortality, reflecting the severity of accelerated atherosclerotic plaque formation in non-dipper hypertension [4].

Although the literature has explored the association between hypertension and atherosclerosis, the role of different blood pressure phenotypes in the development of atherosclerotic disease remains incompletely elucidated [5]. It is particularly important to investigate the potential mechanistic differences between dipper and non-dipper hypertension, especially their contributions to vascular damage and atherosclerosis. Despite the recognition of non-dipper hypertension as a distinct phenotype with higher cardiovascular risk, studies directly linking this pattern to atherosclerosis remain limited. Understanding the differential impacts of these blood pressure patterns on atherosclerosis could provide valuable insights for personalized cardiovascular risk management strategies.

This study aims to address these gaps by focusing on the relationship between dipper and non-dipper hypertension and atherosclerosis, particularly examining coronary artery calcium scores as a measure of atherosclerotic burden. The goal is to provide a clearer understanding of how circadian blood pressure patterns influence the progression of atherosclerosis, ultimately improving cardiovascular risk stratification and management. By investigating the specific differences between non-dipper and dipper hypertension in relation to atherosclerosis, this study seeks to contribute novel insights into the pathophysiological mechanisms linking blood pressure variability to vascular damage and cardiovascular risk.

## 2. Participants and Methods

Between June 2023 and January 2024, a total of 245 patients with a history of hypertension or newly diagnosed hypertension and moderate-to-high cardiovascular risk were recruited from the Cardiology Clinic of Ondokuz Mayıs University Faculty of Medicine. This study was designed as a prospective, single-center study and was approved by the Clinical Research Ethics Committee of Ondokuz Mayıs University on 28 March 2024, under the decision number OMU KAEK 2022/112. “Evaluation of Coronary Artery Calcium Score (CACS) in Dipper and Non-dipper Hypertensive Patients with Moderate and High Cardiovascular Disease Risk”, according to similar studies previously carried out in the literature, the required sample size was determined as a total of at least 75 individuals as a result of the power analysis performed via PASS 11 (Power and Sample Size, version 11, for Windows).

Inclusion criteria were as follows: patients aged ≥40 years; patients with a history of hypertension or newly diagnosed hypertension confirmed clinically; patients with moderate-to-high CVD risk based on a 10-year ASCVD (atherosclerotic cardiovascular disease) risk assessment; patients requiring coronary computed tomography angiography for CACS evaluation; patients who underwent 24-h ABPM (ambulatory blood pressure monitor) for classification into dipper and non-dipper subgroups.

Exclusion criteria were as follows: patients with known secondary hypertension etiologies; patients with chronic kidney disease (CKD) or severe renal dysfunction (e.g., eGFR <30 mL/min/1.73 m²); patients with liver disease; patients with severe endocrine disorders; patients with acute illnesses or infections; pregnant individuals; patients unwilling or unable to comply with 24-h ABPM or CT (computed tomography) scanning; patients aged <40 years.

Patients aged 40 years and older, of both genders, with moderate-to-high cardiovascular risk, and those requiring non-invasive imaging were recorded. The SCORE 2 (systematic coronary risk evaluation 2) and SCORE 2-OP (systematic coronary risk evaluation 2-older persons) scoring systems were used to estimate 10-year cardiovascular risk [6]. Patients with a prior history of hypertension or newly diagnosed hypertension were included in the study. Hypertension was defined according to the latest guidelines: systolic blood pressure (SBP) ≥ 140 mmHg or diastolic blood pressure (DBP) ≥ 90 mmHg based on office blood pressure measurements. Patients were categorized as having dipper hypertension if their nighttime BP dropped by at least 10% compared to daytime values, while those with a nighttime BP decrease of less than 10% or no decrease at all were classified as having non-dipper hypertension. ABPM was also utilized, with hypertension defined as a 24-h mean daytime BP ≥ 135/85 mmHg or nighttime BP ≥ 120/70 mmHg [7]. Blood pressure measurements were conducted using 24-h ABPM (Biomedical Instruments Co., Ltd, BI9800TL Series, Shenzhen, China) devices. Daytime measurements were recorded at 15-mine intervals between 6:00 a.m. and 10:00 p.m., while nighttime measurements were recorded at 30-min intervals between 10:00 p.m. and 6:00 a.m. Patients were instructed to continue their daily routines during ABPM monitoring while avoiding excessive physical activity. Additionally, patients were reminded to log their sleep and wake times, which were crucial for accurately evaluating nighttime BP measurements. Device accuracy was ensured through regular calibration and testing, enhancing the reliability and reproducibility of the collected data.

CACS was measured using a 64-slice multidetector computed tomography (MDCT) scanner (Siemens Somatom Definition AS). All CT scans were performed using a standard protocol, including a low-dose protocol to minimize radiation exposure. The scans were analyzed in two stages to assess both calcium scores and the presence of calcified plaques in the coronary arteries. Calcium scoring was quantitatively calculated using the Agatston method, a validated marker of coronary artery disease severity. The MDCT scanner identified calcium deposits in coronary arteries as bright white spots and measured their intensity in Hounsfield units (HUs). Individual scores were assigned to each coronary artery segment based on calcium intensity: 1 for 1–100 HU, 2 for 101–400 HU, and 3 for >400 HU. The total CACS was the sum of scores across all coronary artery segments. The scores were interpreted as follows: 0 indicated no detectable calcium and low risk; 1–10 indicated minimal calcification (low risk); 11–100 indicated mild calcification (moderate risk); 101–400 indicated moderate calcification (moderate-to-high risk); and >400 indicated extensive calcification and high coronary artery disease risk [8].

Data on patient demographics, including age, gender, height, weight, BMI (body mass index), smoking and alcohol habits, and comorbid conditions, were collected. Patients underwent a thorough anamnesis and physical examination, and eligible individuals were identified. Before the procedure, lipid profile, glycated hemoglobin, complete blood count, kidney function, and liver function were performed. The estimated glomerular filtration rate (eGFR) was calculated using the Modification of Diet in Renal Disease (MDRD) formula. Antihypertensive medications were documented. A comprehensive laboratory testing panel was performed to evaluate various biochemical parameters associated with cardiovascular risk. These tests included CBC (complete blood count), liver and kidney function tests (BUN (blood urea nitrogen), creatinine, and eGFR), HDL (high-density lipoprotein), LDL (low-density lipoprotein) and triglycerides, and glycosylated hemoglobin (HbA1c). All biochemical analyses were conducted using standardized, automated equipment at the Clinical Laboratory of Ondokuz Mayıs University Faculty of Medicine.

Serum glucose, HbA1c, and lipid levels were measured using the Abbott Architect c4000 system, while kidney function markers such as creatinine and BUN were analyzed on the Roche Cobas 8000 platform. All tests adhered to manufacturers’ protocols, ensuring analytical precision and accuracy through routine quality control evaluations.

After the data obtained from the study were coded, they were transferred to a computer and analyzed using SPSS (Version 22 for Windows, SPSS Inc, Chicago, IL, USA) software package. When evaluating the data, continuous variables were expressed as mean ± standard deviation if they were parametric, and as median (minimum and maximum values) if they were non-parametric. Frequency data were expressed as numbers and percentages (%). The normality of all measurement variables was assessed using the “Kolmogorov–Smirnov Test”. In the comparison of frequency data, the “Pearson Chi-square Test” and “Fisher’s Exact Test” were used. Since continuous variables did not follow a normal distribution, the Mann–Whitney U Test was used for comparisons between the two groups. Correlations between variables were analyzed using the “Spearman Correlation Test”. The strength of the relationship was classified based on the correlation coefficient (r): r = 0.00–0.24 as “weak”, r = 0.25–0.49 as “moderate”, r = 0.50–0.74 as “strong”, and r = 0.75–1.00 as “very strong” (Aksakoğlu G. Research Techniques and Analysis Methods in Health, D.E.Ü. Rectorate Printing House, 2nd Edition, İzmir 2006). The relationship between some independent variables and non-dipper HT was evaluated using univariate and multivariate binary logistic regression analysis. To determine the ability of the coronary artery calcium score to differentiate between dipper and non-dipper HT, ROC (receiver operating characteristic) curve analysis was performed. In the ROC analysis, if the area under the curve (AUC) was *p* < 0.05, it was considered that the discriminative ability was statistically significant and a cutoff value was identified. Sensitivity and specificity values were calculated for the cutoff point. A statistical significance level of *p* < 0.05 was accepted for all tests.

## 3. Results

**Figure 1:**
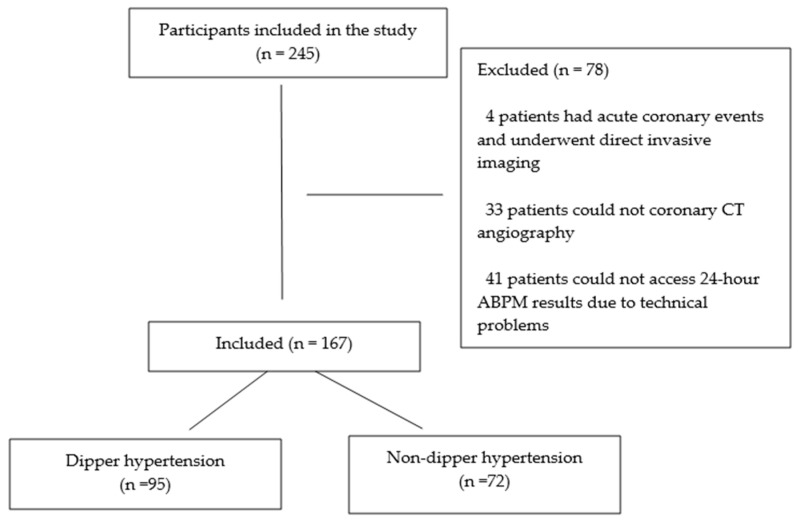
Flowchart of the study population.

The study included 245 patients with moderate and high cardiovascular (CV) risks. Among these patients, four experienced acute coronary events and underwent direct invasive imaging. A total of 33 patients were excluded because they did not undergo coronary CT angiography, and 41 patients were excluded due to technical issues preventing access to their 24-h ABPM results. Ultimately, 167 patients were included in the study. The cohort was divided into two subgroups: 95 patients with dipper HT and 72 with non-dipper HT (Figure 1). The dipper HT group comprised 60 women (63.2%) and 35 men (36.8%), whereas the non-dipper HT group included 28 women (38.9%) and 44 men (61.1%), a statistically significant difference (*p* = 0.002). The mean age was 57 years in the dipper HT group and 62 years in the non-dipper HT group, with a statistically significant difference (*p* = 0.011).

The mean CACS was significantly higher in the non-dipper HT group (93) compared to the dipper HT group (10) (*p* < 0.001). Clinically, patients with non-dipper HT had a higher prevalence of prior coronary artery disease (CAD) compared to those with dipper HT, which was statistically significant (*p* = 0.003). There were no significant differences in baseline laboratory findings between the groups. A history of smoking was significantly more common in the non-dipper HT group (31 patients, 43.1%) than in the dipper HT group (25 patients, 26.3%) (*p* = 0.023). No significant differences were observed between the groups regarding hypertension or statin therapy use (Table 1).

In the correlation analysis of CACS with other variables, significant positive correlations were observed with age, BMI, and HbA1c, while a significant negative correlation was noted with eGFR (Table 2). Higher CACS values were associated with male sex, a history of CAD, diabetes mellitus (DM), and hyperlipidemia, all of which were statistically significant (Table 3).

In univariate analysis, age, male sex, smoking, CAD, CACS, and elevated creatinine were identified as significant risk factors for non-dipper HT (*p* < 0.05). However, in multivariate analysis, only CACS emerged as a significant independent risk factor (*p* = 0.001), while other variables were not significant (*p* > 0.05) (Table 4).

**Figure 2:**
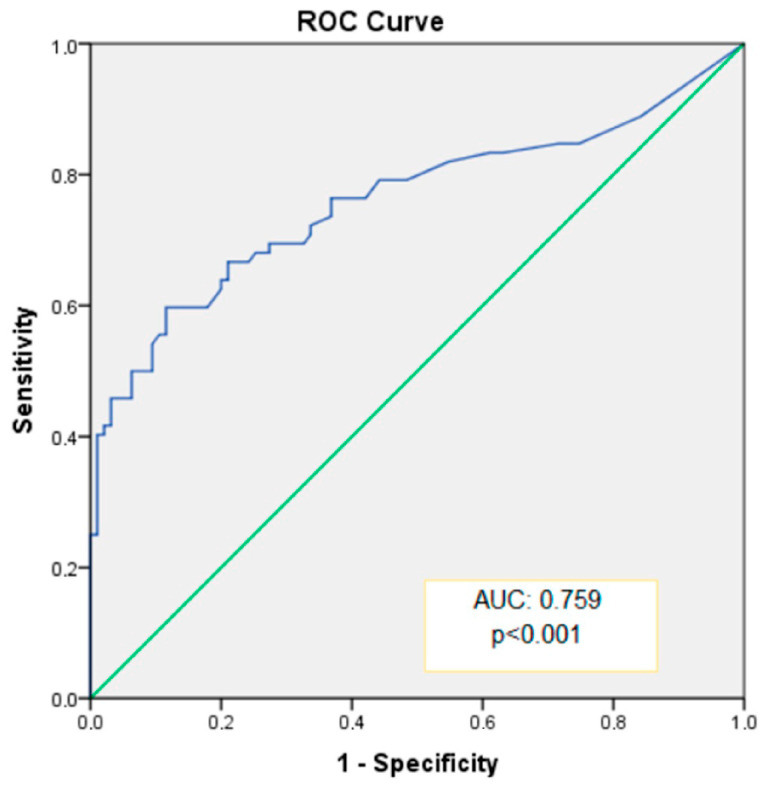
Evaluation of the ability of coronary artery calcium score (CACS) value to distinguish between dipper and non-dipper HT using receiver operating characteristic (ROC) curve.

The ability of CACS to distinguish between dipper and non-dipper HT was assessed, and diagnostic results for specific cutoff values are presented in Table 5. The area under the curve (AUC) for CACS was 0.759 (Figure 2), indicating statistically significant and excellent discriminative capability (*p* < 0.001, 95% CI: 0.680–0.839). When an optimal cutoff value of 35.0 was used for CACS, sensitivity and specificity for predicting non-dipper HT were 66.7% and 78.9%, respectively (Table 5).

## 4. Discussion

This study highlights significant differences in CACS between the dipper and non-dipper hypertension subgroups in patients with moderate-to-high cardiovascular disease risk. Our findings suggest that non-dipper hypertension is associated with a higher CACS and increased CVD risk. While these findings are consistent with the current understanding of hypertension pathophysiology, investigating the underlying mechanisms, integrating them with recent literature, and evaluating their clinical implications are crucial for enhancing our understanding and guiding future interventions.

The higher CACS in the non-dipper group may be explained by several mechanisms. Recent studies have shown that non-dipper hypertension is characterized by elevated nighttime sympathetic activity and sustained renin-angiotensin-aldosterone system (RAAS) activation, leading to persistent endothelial dysfunction and vascular inflammation [9,10]. These mechanisms promote arterial stiffness and calcification, which are directly reflected in elevated CACS. Furthermore, the loss of the physiological nocturnal blood pressure decline in non-dippers exacerbates oxidative stress, accelerating subclinical atherosclerosis [11].

This study identified a statistically significant older mean age in the non-dipper group, consistent with recent findings that age is a critical factor in non-dipping patterns. Aging-related arterial remodeling, increased arterial stiffness, and autonomic dysfunction have been implicated in the progressive loss of nocturnal blood pressure regulation [1]. In addition, the gender disparity, with men exhibiting higher CACS than women, supports the hypothesis that male sex hormones and lower estrogen levels contribute to heightened cardiovascular risk in men. Recent evidence also suggests that postmenopausal women experience a transition toward non-dipping patterns due to hormonal changes, partially mitigating the gender disparity observed in younger populations [12,13].

The observed negative correlation between CACS and eGFR in this study provides valuable insights. As demonstrated by elevated serum creatinine levels or reduced eGFR, impaired kidney function is a well-established risk factor for both hypertension and cardiovascular disease [9]. The relationship between impaired kidney function and increased CACS supports the notion that renal dysfunction likely exacerbates atherosclerosis through mechanisms such as heightened sympathetic activity, endothelial dysfunction, and increased inflammation. In hypertensive patients with a non-dipper pattern, the combined effect of impaired kidney function and sustained hypertension may accelerate the development of coronary artery calcification.

Although smoking rates were higher in the non-dipper group, no significant differences in CACS between smokers and non-smokers were observed. However, smoking remains a critical modifiable risk factor for cardiovascular health, and its role in compounding other risks cannot be overlooked [14].

In this study, the CACS was significantly higher in the non-dipper hypertension group compared to the dipper group. This finding supports the notion that non-dipper hypertension is an important risk factor for coronary artery disease. Previous studies have provided evidence linking non-dipper hypertension to an increased risk of myocardial infarction and stroke [15,16]. Furthermore, a higher number of patients with a history of coronary artery disease were found in the non-dipper group, which increases the clinical significance of the CACS and can be considered an important finding in terms of the prognostic value of cardiovascular events.

In the correlation analysis between CACS and certain parameters in this study, a strong positive relationship was found with age, body mass index (BMI), HbA1c, and creatinine levels. The positive relationship between age and CACS particularly highlights the impact of age on cardiovascular risk factors [17,18]. We found a positive correlation between CACS and HbA1c, which suggests that poor metabolic control, particularly in diabetic patients, contributes to a greater atherosclerotic burden. Elevated HbA1c levels, associated with poor control of diabetes, increase the risk of coronary artery disease [19]. These findings support the adverse effects of diabetes and metabolic disorders on cardiovascular health. Additionally, we observed a negative correlation between CACS and eGFR, highlighting the role of kidney function in the progression of atherosclerosis. Impaired renal function, a common feature in non-dipper hypertension, exacerbates vascular inflammation and oxidative stress, thereby accelerating the calcification of coronary arteries [20,21].

The observation that the statin usage rates were similar between the groups does not imply that statins are ineffective in reducing cardiovascular disease (CVD) risk. Instead, this finding reflects the standard practice of universally prescribing statins to individuals with moderate-to-high cardiovascular risk, regardless of blood pressure dipping status. This approach aligns with current clinical guidelines, which recommend statins as first-line therapy for patients at high cardiovascular risk [22]. It is important to note that statins have well-known pleiotropic effects contributing to cardiovascular protection, such as anti-inflammatory properties, plaque stabilization, and improvement in endothelial function [23,24]. These benefits occur independent of nocturnal blood pressure dipping. Therefore, the lack of difference in statin use between the groups does not diminish their protective role but rather highlights the need for future studies to assess their specific effects within subgroups such as non-dipper hypertension. We acknowledge that our study did not specifically investigate the differing cardiovascular outcomes of statin therapy in dipper and non-dipper hypertensive patients. We suggest that future research should include larger cohorts and longer follow-up periods to evaluate whether the combination of statin therapy with personalized blood pressure management strategies could further enhance cardiovascular protection, particularly in higher-risk non-dipper hypertensive individuals.

This study specifically relates dipper and non-dipper hypertension phenotypes to CACS, a subclinical marker of atherosclerosis. Although previous studies have suggested a link between non-dipper hypertension and higher cardiovascular risk, very few have directly investigated CACS as a marker of this risk. By comparing the two groups of dipper and non-dipper hypertensive patients, we provide a more detailed understanding of how these phenotypes influence the progression of atherosclerosis, as reflected in CACS. This is crucial for improving risk stratification and potentially guiding more personalized treatment strategies for patients with these different hypertension subtypes. The presented study also contributes to understanding which factors (such as smoking, age, BMI, and comorbidities) are associated with higher CACS in non-dipper hypertensive patients. These findings may help clinicians identify individuals at higher risk for cardiovascular events, thus providing more personalized care.

The presented study needs to be evaluated while taking into account some limitations. First, this study has a single-center design, which limits its generalizability. Additionally, as this study was conducted over a specific period, it may be subject to temporal bias. Furthermore, the follow-up period for the patients was short, and long-term outcomes were not assessed. There is no follow-up data to evaluate how CACS, blood pressure patterns, and metabolic parameters change over time in these patients. Long-term follow-up is essential to determine whether patients with higher CACS and non-dipper hypertension are more likely to experience cardiovascular events such as myocardial infarction, stroke, or heart failure. Therefore, larger and multi-center studies are needed to determine the long-term cardiovascular outcomes of dipper and non-dipper hypertension. The 24-h ambulatory blood pressure monitoring used to define the dipper and non-dipper groups resulted in some technical issues, leading to missing data. This has resulted in a lack of knowledge regarding the characteristics of patients who were not included in the study. Additionally, the impact of lifestyle and other environmental factors was not adequately assessed in this study. Future studies with larger and multi-center designs are recommended to overcome these limitations.

## 5. Conclusions

In conclusion, this study elucidates the effects of dipper and non-dipper hypertension on the CACS in patients with moderate-to-high cardiovascular risk. It was found that non-dipper hypertension is associated with a higher CACS, indicating that this group carries a greater cardiovascular risk. In this context, clinical practice recommendations include closer monitoring and the development of appropriate treatment strategies for hypertension management. Future studies should investigate the long-term clinical implications of these findings.

## Figures and Tables

**Table 1:** Comparison of baseline demographic, laboratory, and cardiac evaluations.

Parameters	Dipper HT (*n* = 95)	Non-Dipper HT (*n* = 72)	*p* Value
Age, years	57 (40–79)	62 (44–80)	0.011
Sex, *n* (%)			
Female	60 (63.2)	28 (38.9)	0.002
Male	35 (36.8)	44 (61.1)	
Body mass index, kg/m^2^	26 (21.1–30.6)	26.8 (21.8–32.8)	0.26
Coronary artery calcium score (CACS)	10.0 (0–351)	93.0 (0–920)	<0.001
Smoking, *n* (%)	25 (26.3)	31 (43.1)	0.023
Medical history			
Diabetes mellitus, *n* (%)	23 (24.2)	21 (29.2)	0.47
Stroke/transient ischemic attack, *n* (%)	1 (1.1)	3 (4.2)	0.31
Coronary artery disease, *n* (%)	2 (2.1)	10 (13.9)	0.003
Dyslipidemia, *n* (%)	24 (25.3)	24 (33.3)	0.25
Chronic obstructive pulmonary disease, *n* (%)	7 (7.4)	4 (5.6)	0.64
Laboratory variables			
Creatinine, mg/dL	0.8 (0.4–1.6)	0.93 (0.5–5.1)	0.004
eGFR, mL/min/1.73m^2^	89.86 (35.98–186.21)	88.07 (9.46–148.62)	0.19
Haemoglobin, g/dL	13.6 (9.6–18.2)	14.1 (8.0–16.5)	0.45
HbA1c (%)	5.6 (4.6–8.7)	5.85 (4.5–10.7)	0.13
Triglyceride, mg/dL	142.8 (40.4–975.5)	136.3 (50–515.6)	0.83
HDL cholesterol, mg/dL	49.7 (19.1–134.4)	49.8 (29.1–95.1)	0.94
LDL cholesterol, mg/dL	111.5 (34.6–619.1)	110.0 (19.4–201.8)	0.30
Antihypertensive treatments			
Diuretic, *n* (%)	24 (25.3)	16 (22.3)	0.78
Angiotensin converting enzyme inhibitörs, *n* (%)	16 (16.8)	17 (23.6)	0.51
Angiotensin reseptör blockers, *n* (%)	34 (35.8)	17 (23.6)	0.09
Calcium channel blocker, *n* (%)	24 (25.3)	20 (27.8)	0.81
Minerelocorticoid receptor antogonist, *n* (%)	3 (3.2)	0 (0.0)	0.26
Beta blockers, *n* (%)	24 (25.3)	24 (33.3)	0.45
Alfa blockers, *n* (%)	3 (3.2)	3 (4.2)	0.51
Statin therapy, *n* (%)	20 (21.1)	20 (27.8)	0.12

CACS: coronary artery calcium score; eGFR: estimated glomerular filtration rate; HbA1c: glycated hemoglobin; HDL: high-density lipoprotein; HT: hypertension; LDL: low-density lipoprotein.

**Table 2:** Correlation of CACS with some parameters.

Parameters	r	*p* Value
Age, years	0.559	<0.001
Body mass index, kg/m^2^	0.223	0.004
Haemoglobin, g/dL	0.052	0.507
HbA1c (%)	0.328	<0.001
Triglyceride, mg/dL	0.096	0.216
HDL cholesterol, mg/dL	−0.150	0.052
LDL cholesterol, mg/dL	−0.028	0.724
Creatinine, mg/dL	0.331	<0.001
eGFR, mL/min/1.73 m^2^	−0.226	0.003

CACS: coronary artery calcium score; eGFR: estimated glomerular filtration rate; HbA1c: glycated hemoglobin; HDL: high-density lipoprotein; LDL: low-density lipoprotein.

**Table 3:** Comparison of median CACS according to some parameters.

Parameters		*p* Value
Sex	Male	<0.001
	Female	
Smoking	Never	0.07
	Current	
Diabetes mellitus	Yes	<0.001
	No	
Dyslipidemia	Yes	<0.001
	No	
Chronic obstructive pulmonary disease	Yes	0.36
	No	
Stroke/transient ischemic attack	Yes	0.16
	No	
Coronary artery disease	Yes	<0.001
	No	

**Table 4:** Determination of risk factors for non-dipper hypertension.

Parameters	Univariate			Multivariate		
	Beta (SE)	OR (95% CI)	*p* Value	Beta (SE)	OR (95% CI)	*p* Value
Age, years	−0.03(0.01)	0.96 (0.93–0.99)	0.016	0.004 (0.018)	1.004 (0.96–1.03)	0.835
Sex	0.99 (0.32)	2.69 (1.43–5.06)	0.002	−0.25 (0.44)	0.77 (0.32–1.83)	0.560
CACS	−0.01 (0.003)	0.988 (0.982–0.993)	<0.001	−0.11 (0.003)	0.989 (0.982–0.995)	0.001
Smoking	0.75 (0.33)	2.11 (1.10–4.06)	0.024	−0.27 (0.45)	0.76 (0.31–1.85)	0.552
Coronary artery disease	2.01(0.79)	7.50 (1.58–35.40)	0.011	−0.13 (0.99)	0.87 (0.12–6.17)	0.894
Creatinine	−1.77 (0.77)	0.17 (0.03–0.77)	0.021	−0.9 (0.53)	0.55 (0.19–1.57)	0.267

SE: standard error; CACS: coronary artery calcium score; CI: confidence interval; OR: odds ratio.

**Table 5:** The ability of coronary artery calcium score (CACS) to distinguish between dipper and non-dipper HT.

Cut-Off Value	Sensitivity (%)	Specificity (%)	AUC	SE	*p* Value	%95 CI
27.0	69.4	72.6	0.759	0.041	<0.001	0.680–0.839
31.5	68.1	74.7				
35.0	66.7	78.9				

AUC: area under the curve; CI: confidence interval; SE: standard error.

## Data Availability

The data presented in this study are available on request from the corresponding author.
